# Surprising finding of right-to-left shunt revealed with computed tomography angiography

**DOI:** 10.1007/s12471-020-01402-4

**Published:** 2020-03-10

**Authors:** M. Kardos, M. Sagat, M. Kaldararova, P. Tittel, M. Nosal

**Affiliations:** 1 Department of Functional Diagnostics, Children’s Cardiac Center, Bratislava, Slovakia; 2 Department of Cardiac Surgery, Children’s Cardiac Center, Bratislava, Slovakia

## Answer

**Fig. 1:**
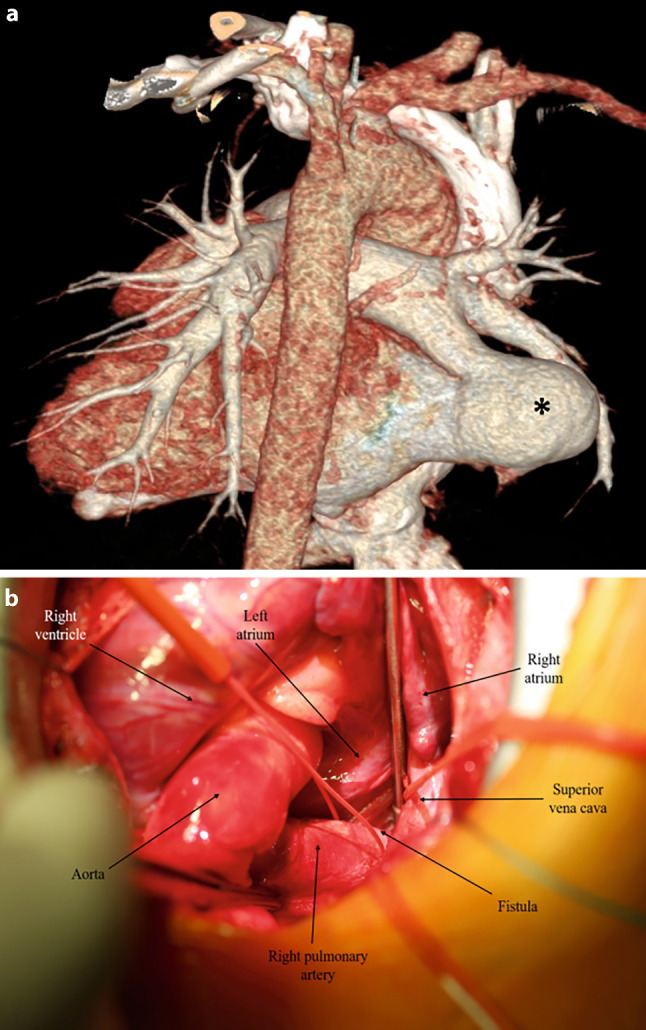
**a** *Asterisk* showing presence of fistula between the right pulmonary artery and the left Atrium. **b** Intraoperative image of surgical ligation of this fistula

Performed computed tomography angiography confirmed the presence of a direct communication between the right pulmonary artery (RPA) and the left atrium via a sac, known as a right pulmonary to left atrium fistula (RPA-LAF) (Fig. 1). No transcatheter closure was planned because of fear of a device embolisation.

The patient underwent open heart surgery with ligation of mentioned fistula. His postoperative course was uneventful and he was discharged home 5 days later. His echocardiogram prior to discharge showed no residual fistula and unobstructed flow in pulmonary artery branches and veins.

Direct communication between the right pulmonary artery and left atrium via a sac represents an unusual variation of a left-to-right shunt. A congenital right pulmonary artery to left atrium fistula usually involves the proximal right pulmonary artery or its lower lobe branch. A fistula between the right pulmonary artery and left atrium may cause cardiac failure in utero. This congenital heart defect can be treated surgically and in selected cases can be performed with transcatheter closure. In fact, there are only about 50 cases reported in the medical literature. Here we present a case of this pulmonary arteriovenous malformation, which was treated surgically [1, 2].
